# LES of Nonlinear Saturation in Forced Turbulent Premixed Flames

**DOI:** 10.1007/s10494-017-9811-4

**Published:** 2017-05-05

**Authors:** Chin Yik Lee, Stewart Cant

**Affiliations:** Department of Engineering, University of Cambridge, Cambridge, UK

**Keywords:** Nonlinear saturation, Turbulent premixed flame, Flame surface density, Flame describing function

## Abstract

The mechanisms for nonlinear saturation of a bluff-body stabilised turbulent premixed flame are investigated using LES with the transported flame surface density (TFSD) approach to combustion modelling. The numerical simulation is based on a previous detailed experimental investigation. Results for both the unforced non-reacting and reacting flows are validated against experiment, demonstrating that the fundamental flow features and predicted flame structure are well captured. Key terms in the FSD transport equation are then used to describe the generation and destruction of flame surface area for the unforced reacting flow. In order to investigate the non-linear response of the unsteady heat release rate to acoustic forcing, four harmonically forced flames are considered having the same forcing frequency (160 Hz) but different amplitudes of 10 *%*, 25 *%*, 50 *%* and 64 *%* of the mean inlet velocity. The flame response is characterised using the Flame Describing Function (FDF). Accurate prediction of the FDF is obtained using the current approach. The computed forced flame structure matches well with the experiment, where effects of shear layer rollup and growth of the vortices on the flame can be clearly observed. Transition to nonlinearity is also observed in the computed FDF. The mechanisms leading to the saturation of the flame response in the higher amplitude case are characterised by inspecting the terms in the FSD transport equation at conditions when the integrated heat release is at its maximum and minimum, respectively. Pinch-off and flame rollup can be seen in snapshots taken at the phase angle of maximum integrated heat release. Conversely, intense vortex shedding and flame-sheet collapse around the shear-layer, as well as small-scale destruction of flame elements in the wake, can be seen in snapshots taken at the phase angle of minimum integrated heat release. The pivotal role of FSD destruction on nonlinear saturation of the flame response is confirmed through the analysis of phase-averaged terms in the FSD transport equation taken at different locations. The phase-averaged subgrid curvature term is found to concentrate in the cusps and downstream regions where flame annihilation is dominant.

## Introduction

Combustion in gas turbines, for example in power plants and aeroengine combustors, produces NO _*x*_ emissions that cause air pollution. To reduce the amount of NO _*x*_, lean premixed combustion is used. Nevertheless, lean operation increases the susceptibility of a combustor to thermoacoustic instabilities. These instabilities may restrict the operating conditions and can severely damage the combustion system. Thermoacoustic instabilities arise when the unsteady heat release is in-phase with the pressure fluctuations [1]. As a result, a feedback loop is formed where chemical energy is fed into the acoustic energy of the combustion chamber. When the acoustic energy gained by the system exceeds the losses from damping processes, the amplitude of the pressure fluctuations grows. In many practical systems, the growth in amplitude is influenced by nonlinear processes, and reaches a saturated state. An understanding of the interaction between flow-field perturbations, acoustic waves and unsteady heat release can aid the design of combustors to avoid the regimes under which this self-reinforcing cycle may reach high fluctuation amplitudes and may be used to determine safe operational regimes or to design control strategies [2–4].

Linear combustion processes generally control the balance between driving and damping, and determine which nonlinear processes emerge to govern the frequency and growth rate of the pressure fluctuations in the combustion chamber. Conversely, nonlinear processes control the finite amplitude dynamics of the oscillations. Hence it is necessary to understand the mechanisms associated with the underlying flow field and flame response to large amplitude perturbations in order to characterise the effects of saturation and predict the limit cycle amplitude of self-excited thermoacoustic oscillation [5, 6]. Nonlinearities can also determine various phenomena such as stability triggering, mode switching and hysteresis [7–9].

Experimental and numerical studies in the past have generally focused on characterising the onset of instability by employing the linear acoustic flame transfer function (FTF) of the system. More recently, experimental studies that serve to characterise the nonlinear flame response of inlet perturbations and identify the amplitude at which saturation occurs have been performed [10]. It was shown that nonlinearities associated with the combustion dynamics could be analysed with a unified framework [7] in which the flame transfer function is replaced by a flame describing function (FDF) that depends on the amplitude of perturbations affecting the flame. A frequency domain stability analysis is then used to yield growth rates and frequencies which depend on the amplitude of the perturbations. Interesting insights on the nonlinear behaviour of acoustically forced flames have been gained through such studies, for example, changes in the flame response due to differences in the burner geometry [9], flame shapes [11, 12] and unsteady flow field [13].

While many experimental studies have confirmed the basic characteristics and saturation amplitude of the flame response to inlet perturbations, the underlying mechanisms for saturation have not been fully explored as yet. There are, however, some efforts to understand the mechanisms that lead to saturation. For example, stabilisation point dynamics were found to affect the nonlinear flame behaviour. Unsteady flame liftoff can reduce the flame area, causing the flame response to saturate at high forcing amplitudes [14]. Saturation of the FDF due to flame quenching was also found to be caused by local extinction and re-ignition [12]. Intense rollup of the flame front has been shown to lead to the generation of vortices from the shear layer that can cause destruction of flame surface area due to the presence of cusps in a bluff-body stabilised flame [10]. Similar observations have been made for a swirled flame, where the downstream displacement stops growing with perturbation amplitude and the flame rolls up into the central recirculation zone [9, 14, 15]. Changes in the swirl velocity fluctuations was found to result in saturation of the flame surface density. Nonlinearities in the flame response can also be effected by kinematic restoration processes [16] in which the extent of flame wrinkling decreases due to flame area destruction [17]. These mechanisms demonstrate that the flame itself is a nonlinear entity and the flame sheet kinematics can play a major role in determining the saturation mechanism of acoustically excited turbulent premixed flames.

A detailed CFD study offers the potential to explore the flow field, and the thermodynamic and kinematic response of the combustion system. In particular, large eddy simulation (LES) has been shown to be capable of predicting the flame dynamics and heat release response for both forced and self-excited thermoacoustic oscillations [18, 19]. LES is therefore adopted here to investigate the behaviour of both the unforced and forced premixed flames. The present work considers the case of a bluff-body stabilised turbulent premixed ethylene-air flame studied experimentally by Balachandran et al. [10]. This case has the advantage of (i) having an acoustically short flame which simplifies extraction of the unsteady heat release rate when inlet forcing is applied; (ii) having extensive data to validate against and (iii) having well controlled acoustic boundaries. In this study, the flame is acoustically forced at the inlet with amplitude up to 64 *%* of the mean flow velocity. The nonlinear flame describing function is measured using a combination of flow-flame diagnostics including OH-PLIF and CH _2_O-PLIF [20], allowing the mechanisms of nonlinearities to be investigated. Rich flame dynamics at different forcing frequencies and amplitudes have been recorded and represented using the flame surface density (FSD). A key observation in this study is the rollup of the flame sheet by convective vortices, which leads to flame annihilation events at high forcing amplitudes. This was found to play an important role in the saturation of the flame response. There have been previous CFD studies for this configuration. Armitage et al. [21] performed a URANS simulation to capture the flame structure in the presence of strong vortex rollup and FDF of the bluff-body premixed flame. A later URANS study by Ruan et al. [22] investigated the effects of chemistry on the forced flame behaviour. They found that while changes in chemical kinetics yield differences in flame length and spatial heat release, its effect on the FDF is small. A LES study by Han et al. [23] using an incompressible solver showed improved results compared to URANS for the gain and phase of the FDF. They also reported that the use of an algebraic combustion model can potentially overpredict the turbulent flame speed and affect the predicted FDF magnitude.

In this paper, the turbulent premixed flame [10] is investigated using the transported flame surface density (TFSD) model [24, 25]. The derivation of the TFSD model is based on theoretical considerations for a propagating surface [26, 27]. The transported FSD model does not invoke the assumptions behind most algebraic FSD models in which subgrid scale flame area production and destruction are taken to be in equilibrium, but instead solves a balance equation for FSD that accounts separately for the strain rate, propagation and curvature effects within the flame. This formulation offers key advantages for the current numerical study. Firstly, in the context where the flame dynamics are governed by flame front kinematics [10], the TFSD model is shown to have advantages over other models in the description of the flame surface evolution where the level of sub-grid wrinkling is high and the flame propagation is highly unsteady [28, 29]. Secondly, the terms in the TFSD equation explicitly determine the generation and destruction rate of the flame surface area on a local basis, and can be used to identify the mechanisms that contribute to the saturation in heat release response.

The key aim of this work is to demonstrate the ability of the TFSD model to represent the turbulent premixed flame in the experiment [10], capture the FDF and describe the saturation mechanisms of combustion instabilities. The paper is organised as follows. Validation of the current LES approach for both the unforced non-reacting and reacting flows will be first presented. Next, individual terms in the FSD transport equation are presented to illustrate the importance of non-equilibrium effects on the flame surface area evolution in localised regions without acoustic forcing. Four cases for the forced reacting flows are then considered. Sinusoidal forcing is applied to the inlet velocity such that the frequency and amplitude of forcing can be independently varied. The forced flame dynamics are illustrated and compared against experiment, and the FDF of both these cases are computed. The key saturation mechanism observed in the experiment at high forcing amplitude, primarily the flame front kinematics that has led to the generation and destruction of flame surface area during the forcing cycle are then described in detail.

## Numerical and Modelling Framework

The governing equations for turbulent combustion LES are the Navier-Stokes equations for mass, momentum, and energy conservation. The Dynamic Smagorinsky model [30] is used to solve for the subgrid stresses *τ*_*ij*_.

The reaction progress variable *c* is used to describe the thermochemistry of a turbulent premixed flame. The reaction progress variable takes a value of zero in the reactant and unity in the product

1 $$ c=\frac{Y_{f}-Y_{fr}}{Y_{fp}-Y_{fr}} $$

where *Y*_*f*_ is the mass fraction of the fuel, and *Y*_*fr*_ and *Y*_*fp*_ represent the mass fraction of fuel in the reactants and products, respectively. The Favre-filtered transport equation for the reaction progress variable is taken as

2 $$\begin{array}{@{}rcl@{}} \frac{\partial{\bar{\rho}}{\tilde{c}}}{\partial{t}}+\frac{\partial{(\bar{\rho}}\tilde{u_{i}}\tilde{c})}{\partial{x_{i}}}+\frac{\partial}{\partial x_{i}}[\overline{\rho}(\widetilde{u_{i}c}-\tilde{u}_{i}\tilde{c})]=\overline{\frac{\partial}{\partial{x_{i}}}\left( \rho D \frac{\partial c}{\partial x_{i}}\right)}+\overline{\dot{\omega}} \end{array} $$

The terms in Eq. 2 in order from left to right are the unsteady term, convection term, subgrid flux of reaction progress variable, molecular diffusion term and chemical reaction rate. The quantity *D* is the progress variable diffusivity. The subgrid scalar flux term is modelled using a gradient transport hypothesis [31]

3 $$ \frac{\partial}{\partial x_{i}}[\overline{\rho}(\widetilde{u_{i}c}-\tilde{u}_{i}\tilde{c})]=\frac{\partial}{\partial x_{i}}\left( -\frac{\mu_{t}}{\text{Sc}_{sg}}\frac{\partial \tilde{c}}{\partial x_{i}}\right) $$

where *μ*_*t*_ is the turbulent viscosity and Sc_*sg*_ is the subgrid scale Schmidt number. The combined reaction rate and molecular diffusion rate is modelled as [24]:

4 $$ \overline{\frac{\partial}{\partial{x_{i}}}(\rho D \frac{\partial c}{\partial x_{i}})}+\overline{\dot{\omega}}= \overline{(\rho S_{d})}_{s}{\Sigma}_{gen}=\rho_{u}S_{L}{\Sigma}_{gen} $$

where *ρ*_*u*_ the density of the unburnt mixture, *S*_*L*_ is the laminar flame speed and Σ_*gen*_ is the generalized FSD given by ${\Sigma }_{gen}=\overline {|\nabla c|}$ [32]. The transport equation for Σ_*gen*_ is expressed as [24, 25]

5 $$\begin{array}{@{}rcl@{}} \frac{\partial{{\Sigma}_{gen}}}{\partial{t}}+\frac{\partial(\tilde{u}_{i}{\Sigma}_{gen})}{\partial{x_{i}}}&=&-\frac{\partial}{\partial x_{i}}[\overline{(u_{i})}_{s}-\tilde{u}_{i}]{\Sigma}_{gen} +\overline{\left( S_{d}\frac{\partial N_{i}}{\partial x_{i}}\right)_{s}}{\Sigma}_{gen}\\ && -\frac{\partial}{\partial x_{i}}[\overline{(S_{d}N_{i})}_{s} {\Sigma}_{gen}]+\overline{\left( (\delta_{ij}-N_{i}N_{j})\frac{\partial u_{i}}{\partial x_{j}}\right)}_{s}{\Sigma}_{gen} \end{array} $$

where the terms on the RHS of Eq. 5 represent subgrid convection, curvature, propagation and tangential strain rate. The subgrid convection term accounts for scalar transport due to turbulent fluctuations and changes in velocity across the flame, and takes the form [33]:

6 $$\begin{array}{@{}rcl@{}} \frac{\partial}{\partial x_{i}}[\overline{(u_{i})}_{s}-\tilde{u}_{i}]{\Sigma}_{gen}&=&-\frac{\partial}{\partial x_{i}}\left( \frac{\nu_{t}}{\text{Sc}_{\Sigma}}\frac{\partial {\Sigma}_{gen}}{\partial x_{i}}\right)-\frac{\partial}{\partial x_{i}}[(c^{*}-\tilde{c})\tau S_{L}\overline{(N_{i})}_{s}{\Sigma}_{gen}] \end{array} $$

The turbulent Schmidt number for the FSD Sc_Σ_ is taken to be equal to Sc_*sg*_ (3) [34]. The surface averaged flame normal $\overline {(N_{i})}_{s}$ is expressed as

7 $$ \overline{(N_{i})}_{s}=-\frac{1}{{\Sigma}_{gen}}\frac{\partial \overline{c}}{\partial x_{i}} $$

The surface averaged displacement speed $\overline {(S_{d})}_{s}$ is modelled as

8 $$ \overline{(S_{d})}_{s}=S^{\prime}_{L}(1+\tau c^{*}) $$

where *τ* is the heat release parameter, *c* ^∗^ is the progress variable at a given isosurface and $S^{\prime }_{L}$ is the modified flame speed incorporating the effects of straining and curvature that are dominant within the thin reaction zone regime [35].

The contribution of the propagation and curvature terms in Eq. 5 can be decomposed as [24, 25]:

9 $$\begin{array}{@{}rcl@{}} -\frac{\partial}{\partial x_{i}}[\overline{(S_{d}N_{i})}_{s} {\Sigma}_{gen}]+\overline{\left( S_{d}\frac{\partial N_{i}}{\partial x_{i}}\right)_{s}}{\Sigma}_{gen} =P_{mean}+C_{mean}+C_{sg} \end{array} $$

where *P*_*mean*_ and *C*_*mean*_ are the resolved contributions of the propagation and curvature terms. The term *C*_*sg*_ is the subgrid curvature.

The strain rate term describes the strain induced by the surrounding fluid on the flame and can be expressed as

10 $$ \overline{\left( (\delta_{ij}-N_{i}N_{j})\frac{\partial u_{i}}{\partial x_{j}}\right)}_{s}=S_{mean}+S_{hr}+S_{sg} $$

where *S*_*mean*_ is the resolved strain rate and *S*_*hr*_ is the strain rate due to heat release. The subgrid strain rate *S*_*sg*_ includes the subgrid contribution of strain rate effects due to both turbulence and heat release.

The modelled transport equation for FSD [24, 25] takes the form below when the expression for each term is incorporated

11 $$\begin{array}{@{}rcl@{}} \frac{\partial{{\Sigma}_{gen}}}{\partial{t}}\,+\,\frac{\partial(\tilde{u}_{i}{\Sigma}_{gen})}{\partial{x_{i}}} \!&=&\!\frac{\partial}{\partial x_{i}}\left( \frac{\nu_{t}}{Sc_{\Sigma}}\frac{\partial {\Sigma}_{gen}}{\partial x_{i}}\right)\,+\,[\delta_{ij}\,-\,\overline{(N_{i}N_{j})}_{s}]\frac{\partial \tilde{u}_{i}}{\partial x_{j}}{\Sigma}_{gen} \,+\,{\Phi}{\Gamma} \frac{\sqrt{k}}{\Delta}{\Sigma}_{gen} \\ &&\! -\frac{\partial}{\partial x_{i}}(\overline{(S_{d})}_{s}\overline{(N_{i})_{s}}{\Sigma}_{gen}) \,+\,\overline{(S_{d})}_{s}\frac{\partial \overline{(N_{i})}_{s}}{\partial x_{i}}{\Sigma}_{gen} \,-\,\alpha\beta S_{L}\frac{{\Sigma}_{gen}^{2}}{1-\overline{c}} \end{array} $$

The terms on the RHS of Eq. 11 are the subgrid convection, resolved strain rate *S*_*mean*_, subgrid strain rate *S*_*sg*_, resolved contributions of propagation *P*_*mean*_, resolved contribution of curvature *C*_*mean*_ and subgrid curvature *C*_*sg*_. The term $\overline {(N_{i}N_{j})}_{s}$ is modelled as $\overline {(N_{i}N_{j})}_{s}=\overline {(N_{i})}_{s}\overline {(N_{j})}_{s}+1/3\delta _{ij}[1-\overline {(N_{k})}_{s}\overline {(N_{k})}_{s}]$. The term Φ is a model constant that takes a value of unity, Γ is an efficiency function which can be described using the Intermittent Net Flame Stretch (ITNFS) model [36] and the subgrid-scale turbulent kinetic energy *k* is estimated using the Dynamic Smagorinsky model according to the relation *k*_Δ_ = *ν*_*t*_/(*C*_*dy*_Δ)^2^, where *C*_*dy*_ is the model constant. The term $\alpha = 1-\overline {(N_{i})}_{s}\overline {(N_{i})}_{s}$ in *C*_*sg*_ is the resolution factor that tends to zero under fully resolved conditions and *β* is a model constant that takes a value of unity. This value was selected through a sensitivity study, and was found adequate for the current configuration.

Due to the presence of walls, special treatment for the TFSD model is required. Different quenching models have been proposed, for example by solving for the transport equation of FSD in the wall regions [37] or by simply including quenching effects on the flame through empirical expressions [38]. Here, the quenching model proposed by Catlin and Lindstedt [39] is adopted. The model suppresses the reaction rate when the progress variable falls below a quenching value for the reaction progress variable $\tilde {c}_{q}$, corresponding to a quenching temperature of $T_{q}=\tilde {c}_{q}(T_{p}-T_{r})$. In the computations, the reaction is quenched when the temperature is below *T*_*q*_, which is specified at 350 K. A similar strategy was adopted by Tagermman and Keppler [40].

The TFSD model is implemented in the open source CFD toolkit OpenFOAM [41]. The equations are discretised in space using a central differencing scheme which is second-order accurate in smooth regions of the solution and is flux limited in near steep gradients in order to guarantee boundedness. A second-order backward time marching scheme is employed to account for transient effects. The system of equations is solved using the PISO pressure correction algorithm [42].

## Configuration and Operating Conditions

**Fig. 1:**
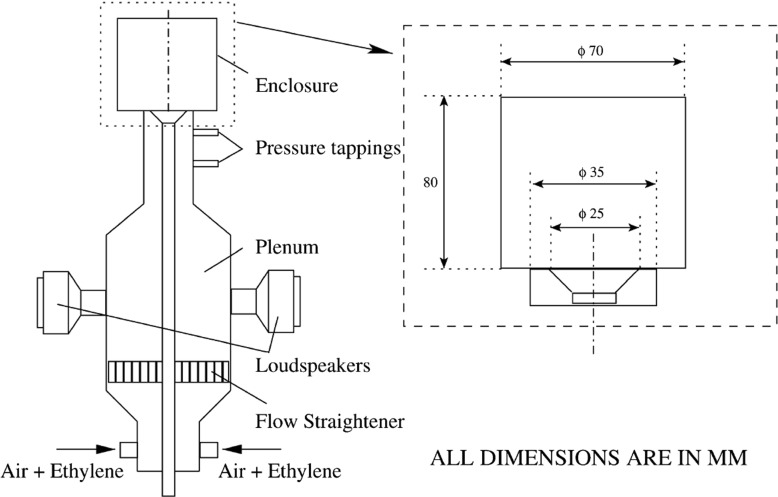
Experimental setup for the bluff-body enclosed premixed flame [10]

The experimental setup [10] is shown in Fig. 1. The rig consists of a long circular duct of internal diameter 35 mm with a conical bluff-body of diameter *d* equal to 25 mm. The angle of the bluff-body is 45 degrees (half angle), giving a blockage ratio of 50 *%*. The enclosure is a 80 mm long quartz cylinder with internal diameter 70 mm to provide optical access for measurements of the flame structure. The region upstream of the rig consists of a plenum chamber and loudspeakers to facilitate acoustic forcing. The time-averaged bulk inlet velocity *U*_*b*_ of the flow is 10 m/s. This gives a Reynolds number Re = *U*_*b*_ *d*/*ν* of 16000, where *d* is the bluff-body diameter and *ν* is the kinematic viscosity. The short enclosure of the combustor provides a resonance for the longitudinal acoustic mode at around 1000 Hz, which is well above the frequency of the forcing. Gaseous ethylene is supplied far upstream of the combustor at a distance of approximately 2 m from the inlet to the combustion enclosure. This allows sufficient time for mixing, thus resulting in a homogenously premixed ethylene-air mixture. The inlet forcing is applied via two loudspeakers, where the amplitude *A* and frequency *f* of the forcing signals are varied independently.

In the numerical simulation, the full enclosure extending up to 30 mm upstream of the bluff-body is simulated, allowing for the flow development. A multi-block mesh is constructed using two different mesh configurations with 3 and 4 million cells. For the dense mesh, there is refinement around the shear layer of the flame stabilisation regions close to the annular inlet. The numbers of cells are approximately 95 across the radius, 200 across the axial direction and 200 along the circumferential direction. A total of 25 cells was used across the annular opening near the bluff body of 5 mm. For these meshes, the ratio of the cell size to the laminar flame thickness lies within the range of 1–5. Mesh independence tests were carried out to confirm the capability of the numerical approach to capture steep gradients of reaction progress variable.

An inlet velocity of 5.15 m/s is prescribed such that the mass balance is satisfied between the inlet of the computational domain and the location where the flow enters the enclosure at the annular opening to achieve a mean velocity of 10 m/s. The temperature at the inlet is fixed at 288 K and the progress variable is set to zero as the inlet contains premixed ethylene-air mixture. The equivalence ratio is *ϕ* = 0.55. The inflow turbulence is prescribed using the Synthetic Eddy Method (SEM) [43] with an amplitude of 5 *%* of the mean velocity. The solid walls are treated as adiabatic and impermeable. A wave-transmissive boundary condition [44] is employed at the outlet. This allows the acoustic waves to leave the domain and enables the combustion chamber to act as an amplifier in the forced reactive flow studies.

For these studies, an adaptive timestep in the range of 1 − 2 × 10^−7^ s has been used. These values ensure a Courant–Friedrichs–Lewy (CFL) number less than 0.1 everywhere in the domain. The LES simulations are run for at least 30 characteristic time periods *τ*_*b*_ = *d*/ *U*_*b*_ before the statistics are collected. The collected data are gathered over 40 *τ*_*b*_ to obtain the results for the mean and second moment quantities.

## Results and Discussion

### Unforced non-reacting and reacting flows

**Fig. 2:**
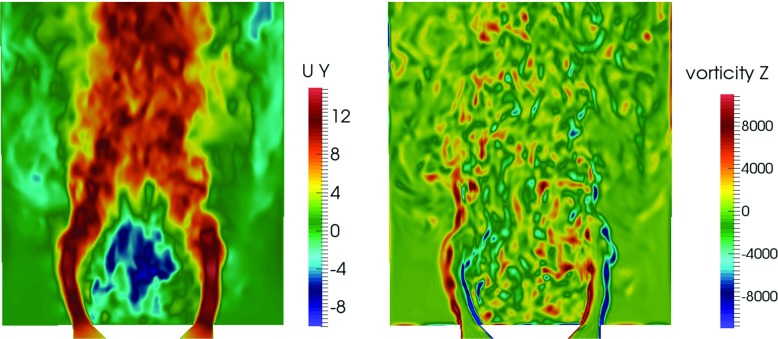
Instantaneous axial velocity (*left*) and radial component of vorticity (*right*)

Before considering the reacting flow, a cold flow validation is performed to assess the mesh resolution, numerical discretisation and subgrid-scale models for LES. Snapshots of the instantaneous axial velocity and the radial component of the vorticity are shown in Fig. 2. The high velocity annular jets, the reverse flow in the central recirculation zone and the outer recirculation zone are evident. The vorticity contour suggests that Kevin-Helmholtz instabilities occur along the separated shear layer, as well as the presence of vortex merging and growth. At the end of the shear layer, some degree of vortex shedding can be observed.

**Fig. 3:**
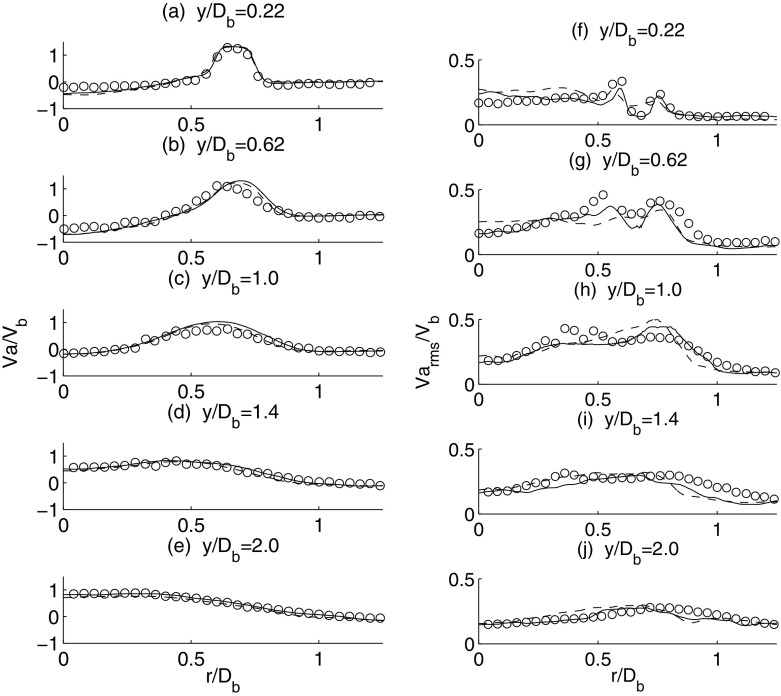
Time-averaged mean axial velocity (*left*) and time-averaged rms axial velocity (*right*). Experiment: *circles*, LES: *solid line* (dense mesh) and *dashed line* (coarse mesh)

The mean and root mean square (rms) of the axial velocity profiles from LES are compared with the experimental data. Figure 3 presents the results for the the mean (a–e) and rms (f–j) axial velocity profiles. The mean and rms velocities are normalised against the inlet velocity *V*_*b*_ and the radial distance against the bluff-body diameter *D*_*b*_. The velocity profiles are obtained at five different locations in the experiment corresponding to *y*/*D*_*b*_ of 0.22, 0.62, 1.0, 1.4 and 2.0 from the annulus, where *y* is the axial distance. Good agreement with the experiment is obtained for the mean axial velocity using both the dense (4 million cells) and coarse (3 million cells) grids. The key flow features including the flow reversal in the recirculation zone (Fig. 3a, b), jet inlet (Fig. 3a–c) and flow expansion (Fig. 3d, e) of the isothermal flow are well captured. Comparable predictions in terms of the rms velocity profiles are also obtained, demonstrating the adequacy of the subgrid-scale modelling to represent the unresolved flow features. Nevertheless, the profiles of the rms velocities are clearly more sensitive to the mesh resolution. The dense mesh is chosen for the reacting flow simulation.

**Fig. 4:**
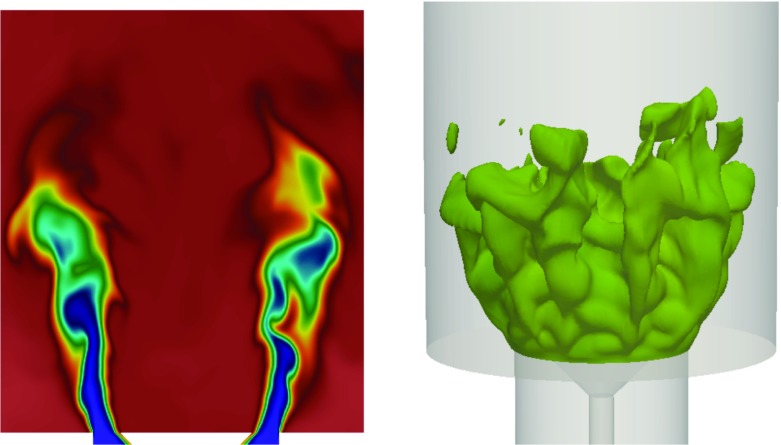
Instantaneous temperature contours (*left*) and isosurfaces of $\tilde {c}=0.5$ (*right*) of the flame

The reacting flow at *ϕ* = 0.55 is then established using the TFSD model as described above. The instantaneous temperature contour is presented in Fig. 4. To complement the 2D view, the iso-surface of the Favre-filtered progress variable $\tilde {c}=0.5$ is displayed in the right column of Fig. 4. The snapshots are obtained at time *t* = 100 ms after the initiation of the simulation where a quasi-steady flame structure has been achieved. The flame spreads widely, and the tip of the flame brush is positioned close to the wall of the enclosure. The flame extends about twice the bluff-body width in the axial direction, consistent to that observed in the experiment [10] and previous LES study [23]. It can also be seen from the progress variable isosurface in Fig. 4 that the flame front is highly corrugated.

**Fig. 5:**
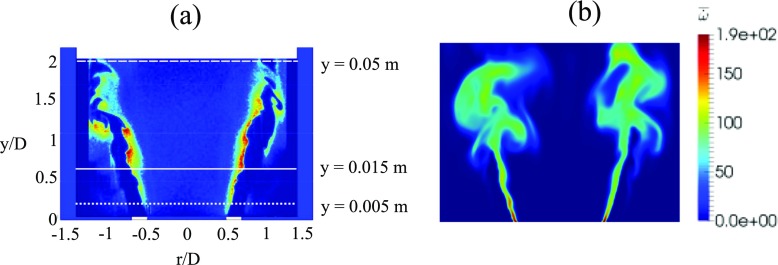
Instantaneous OH-PLIF snapshot from the experiment [10] (**a**) and instantaneous reaction rate contours from LES (**b**)

**Fig. 6:**
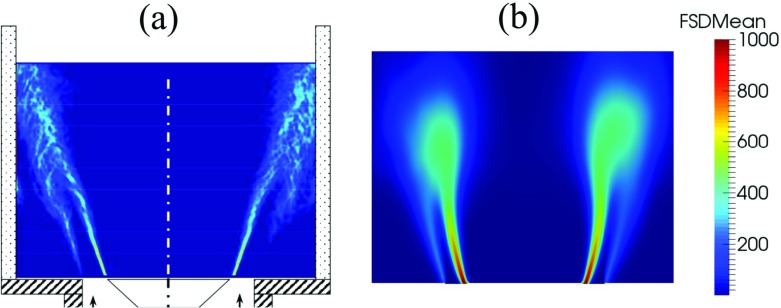
Time-averaged FSD from the experiment [10] and time-averaged FSD contours from LES (**b**)

Figure 5 shows an OH-PLIF image from an instance in the experiment (a) and the instantaneous reaction rate contour from LES (b). From the instantaneous OH-PLIF experimental image (a), it can be seen that the flame is primarily anchored on the inner shear layer formed by the central recirculation zone generated by the bluff-body, with some flame elements weakly stabilised on the outer shear layer associated with the side recirculation zones. Localised regions with high OH intensity can be observed downstream of the shear layer where the flame is highly corrugated. A high heat release rate is expected in these regions due to an increase in the flame area caused by the interaction of vortices with the flame. Key features observed in the experiment are captured in LES. The flame structure indicated by the reaction rate contour (b) exhibits a close resemblance to the experimental OH-PLIF snapshot, where both the flame angle and flame length are well predicted. The flame is also found to be highly wrinkled in the downstream region. The time-averaged FSD magnitude from the experiment and from the TFSD model are also presented in Fig. 6a and b respectively. The time-averaged FSD from LES (b) indicates that locations with high FSD match well with the experiment (a).

**Fig. 7:**
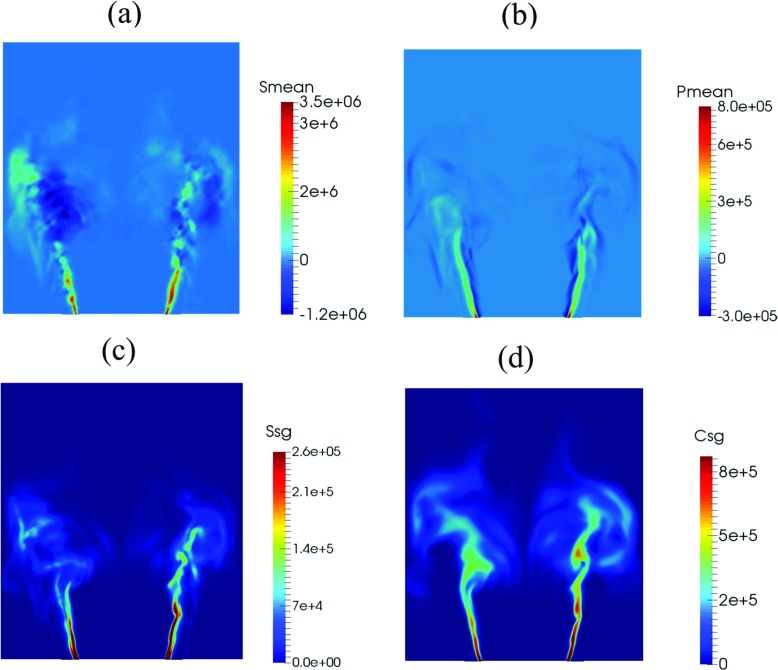
**a** Resolved straining, **b** resolved propagation and curvature, **c** subgrid straining and **d** subgrid curvature from the TFSD equation

Additional physical insight into the flame can be obtained by analysing the individual terms in the transport equation of the TFSD model (11). Figure 7 depicts (a) resolved straining, (b) resolved propagation and curvature, (c) subgrid straining and (d) subgrid curvature. As shown in Fig. 7a, the resolved strain attains a large positive value along the inner shear layer close to the bluff-body, indicating high FSD generation in this region. At the edges of the flame brush downstream of the shear layer, both positive and negative values are present, suggesting that the flame front undergoes both compressive and extensive straining. In Fig. 7b, the resolved propagation and curvature term is seen to be low on the products side of the inner shear layer and high towards the reactants. This term takes positive values in the reactants and negative values in the products. Figure 7c shows high values of subgrid strain in the flow separation region at the edges of the bluff-body where the flame is highly stretched, and along the inner shear layer where the flame stabilises. The subgrid curvature term (Fig. 7d) has a similar trend but with a higher magnitude in the shear layer and at the downstream region close to the wall where flame-vortex interaction occurs.

**Fig. 8:**
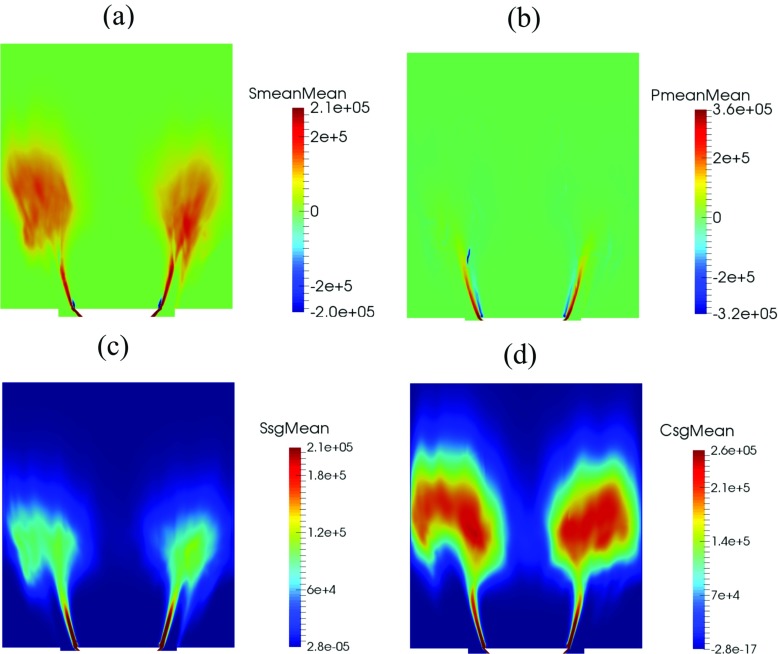
Time-averaged values of (**a**) resolved straining (**b**) resolved propagation and curvature (**c**) subgrid straining and (**d**) subgrid curvature terms from the TFSD model

**Fig. 9:**
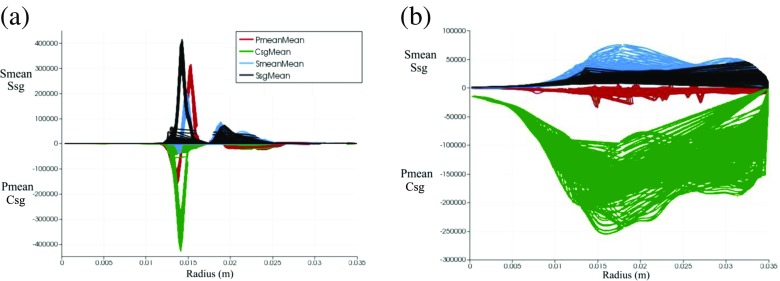
Time-averaged values of resolved straining (*blue*) resolved propagation and curvature (*red*) subgrid straining (*black*) and subgrid curvature (*green*) terms from the TFSD model plotted in **a** plane 1 and **b** plane 2. Plane 1 corresponds to y = 0.005 m (indicated by dotted white line in Fig. 5a) and plane 2 corresponds to y = 0.05 m (indicated by dashed white line in Fig. 5a)

The time-averaged contours of individual terms in the FSD transport equation are presented in Fig. 8. These contours show that both flame area generation and destruction occur primarily in the flow separation region around the flameholder lip and in the shear layer. Activities associated with the flame surface evolution in the subgrid-scales are also found to be prevalent farther downstream, as evidenced by the time-averaged subgrid straining (c) and curvature (d) terms. In the downstream region, the value of the time-averaged resolved flame propagation and curvature term (b) is small. The time-averaged values for both resolved and subgrid strain rate and curvature terms taken at planes 1 and 2 are plotted in Fig. 9 at different radial distance from the centreline. Plane 1 corresponds to the shear layer at y = 0.005 m (indicated by dotted white line in Fig. 5a) and plane 2 corresponds to the flame region farther downstream at y = 0.05 m (indicated by dashed white line in Fig. 5a). Some key observations can be made for each terms in FSD transport equation taken in plane 1. Firstly, values for the resolved terms are smaller than the subgrid terms. This is expected as smaller vortical structures affecting the subgrid flame surface area are present in the separated shear layer. Secondly, the resolved strain rate (blue) and resolved propagation (red) terms can contribute to both generation and destruction of FSD. The resolved strain rate provides a net flame area generation. Thirdly, the subgrid FSD destruction and generation are nearly in equilibrium, as indicated by the similar magnitudes of the subgrid straining (black) and curvature (green) terms. In plane 2, it is clear the magnitude of the subgrid curvature (green) term is far greater than the subgrid straining (black) term. This highlights that flame surface area destruction in the small-scales is dominant in the downstream region.

### Forced premixed flame

The forced reactive cases are simulated by imposing velocity fluctuations on the mean velocity at the inlet using the form

12 $$ u_{inlet}(t)=\bar{u}_{inlet}[1-A\sin{(2\pi ft)}] $$

where *f* is the forcing frequency and *A* is forcing amplitude. Four cases corresponding to forcing amplitude *A* = 0.1, 0.25, 0.5 and 0.64 at forcing frequency *f* = 160 Hz are considered in this work. Complex saturation behaviour has been observed and reported at conditions above *A* = 0.2 [10]. While it is possible to apply inlet forcing methods that allow multiple amplitudes to be excited at once, including ‘sum-of-sines’ forcing [45, 46] or ‘multi-mode’ perturbations [47], these methods are not used in the current work to avoid triggering the underlying nonlinearities of the flame that potentially excite higher harmonics and lead to the interactions of different acoustic modes.

**Fig. 10:**
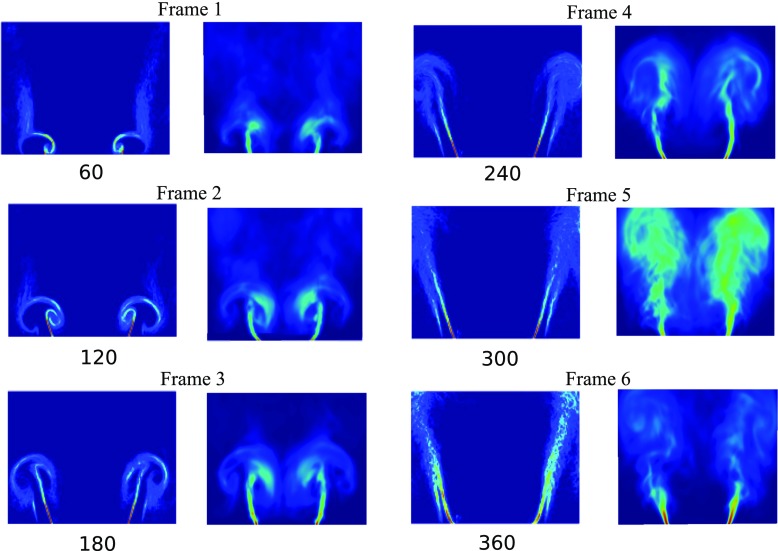
Comparison of phase-averaged experimental FSD and numerical FSD at different six phase angles for *f* = 160 Hz and *A* = 0.64

**Fig. 11:**
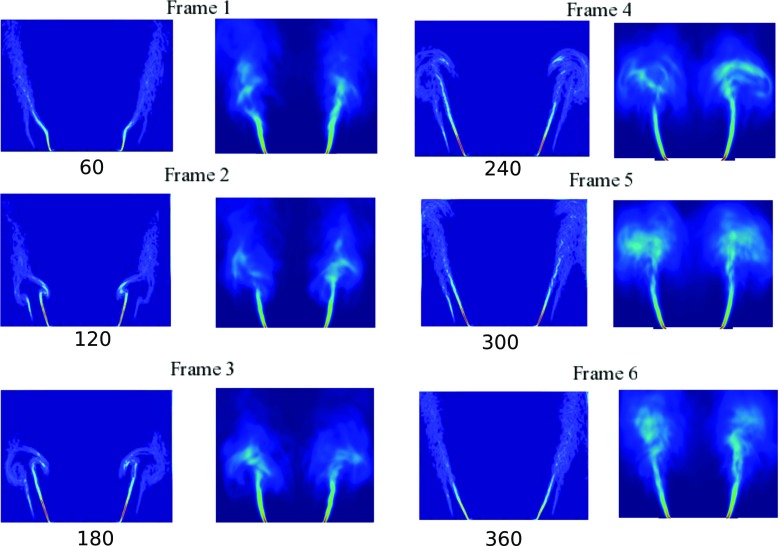
Comparison of phase-averaged experimental FSD and numerical FSD at six different phase angles *f* = 160 Hz and *A* = 0.25

Figure 10 shows the evolution of the flame described using the phase-averaged FSD at six different phase angles (frames 1–6) throughout the forcing cycle for *f* = 160 Hz and *A* = 0.64. For each frame, the left column corresponds to the experimental snapshots and the right column corresponds to the LES results. The images show that deformations of the flame base are evident and the shear layers roll up to form a counterrotating vortex pair during the forcing cycle. The rolled-up flame front grows in size (phase angles 60–180°), convects with the flow and impinges on the side walls of the combustor (phase angles 240–300°). Once the vortex is convected farther downstream, the flame straightens and a new vortex is formed close to the lip of the flameholder (phase angles 360–60°). The phase-averaged FSDs for forcing amplitude *A* = 0.25 at the same phase angles are shown in Fig. 11 in a similar manner. It can be seen that for *A* = 0.25, changes in the flame dynamics involving the rollup of the flame, and interaction of the flame with the wall remain evident. Yet, the extent of rollup decreases and the flame surface is less curved compared to the case of *A* = 0.64. The shortening of the flame is also less prominent. Both Figs. 10 and 11 demonstrate that the phase-averaged FSD results from LES exhibit good agreement with the experiment, where the structure of the flame at different phase angles of the forcing cycle is well captured.

**Fig. 12:**
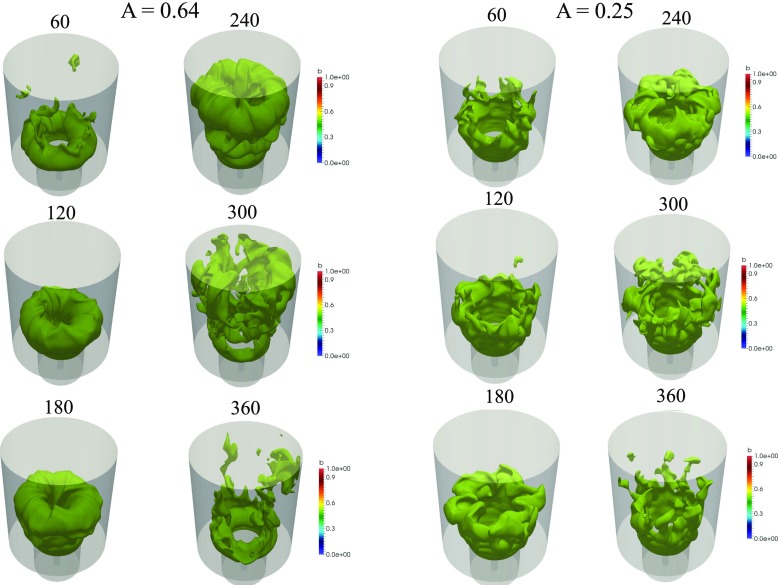
Isosurface of reaction progress variable at $\tilde {c}=$ 0.5 at six different phase angles for *A* = 0.64 (*left*) and *A* = 0.25 (*right*) at *f* = 160 Hz

The instantaneous isocontours of $\tilde {c}=0.5$ at the same phase angles shown in Figs. 10 (*A* = 0.64) and 11 (*A* = 0.25) are presented in Fig. 12. For *A* = 0.64, the vortex formation can be seen clearly and is characterised by the bulging flame structure close to the flameholder. The vortex grows in size and reaches its maximum size at phase angle 120°. At 240°, the flame elongates as the vortex convects downstream. The flame attains its maximum length and some interaction of the flame with the walls occurs. At phase angle 300°, extensive corrugation and tearing of the flame sheet are evident. The detached flame elements eventually leave the combustion chamber and a new cycle repeats itself again. For *A* = 0.25, the global structure of the flame is less coherent compared to *A* = 0.64. In particular, the flame still exhibits some unsteadiness observed under unforced condition, which is manifested by variations in the azimuthal structure characterised by the petal-like structures along the outer shear layer throughout the forcing cycle.

**Fig. 13:**
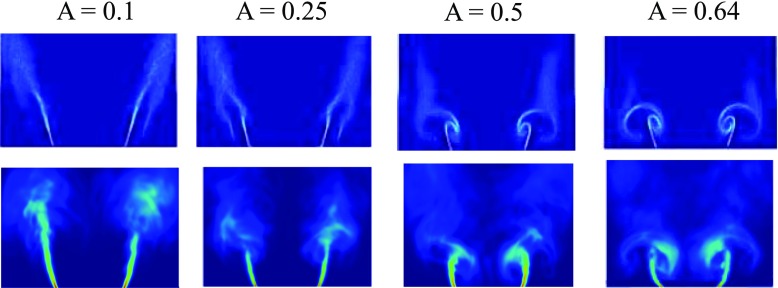
Comparison of phase-averaged experimental FSD (*top*) and numerical FSD (*bottom*) at phase angle 120° for *A* = 0.1, 0.25, 0.5 and 0.64

The averaged FSD images for the four different forcing amplitudes considered at the phase angle when the integrated heat release is minimum (120°) are compared in Fig. 13. The top rows are the experiment snapshots and the bottom rows are the LES results. The numerical results are consistent with the experimental observation. It is found that the flame surface is increasingly arched as forcing amplitude increases. The mushroom-like structure of the flame and cusps at the edges of the flame are evident both in the experiment and CFD at high forcing amplitude especially at *A* = 0.5 and 0.64. Also, the flame length has decreased due to the presence of vortex rollup. These are key features that control the nonlinearities of the flame response.

The heat release rate fluctuation is important in quantifying the heat release response of the flame. In the framework of the TFSD model, the integrated heat release is computed by integrating the reaction rate source term.

13 $$\begin{array}{@{}rcl@{}} Q=H_{f}\int\omega dV=H_{f}\int\rho_{u}S_{L}{\Sigma} dV \end{array} $$

where *H*_*f*_ is the heat of formation, *ρ*_*u*_ is the density of the unburnt mixture, *S*_*L*_ is the laminar flame speed and Σ is the FSD. As discussed previously [22, 23], the integration area for the experiment was based on a measurement window of length 55 mm, as opposed to the full combustion chamber of length 80 mm. Nevertheless, in the previous LES study by Han et al. [23], the difference between the global heat release obtained using these measurement windows was found to be small and the heat release obtained from the total measurement window was therefore used. Here, we follow the same procedure.

**Fig. 14:**
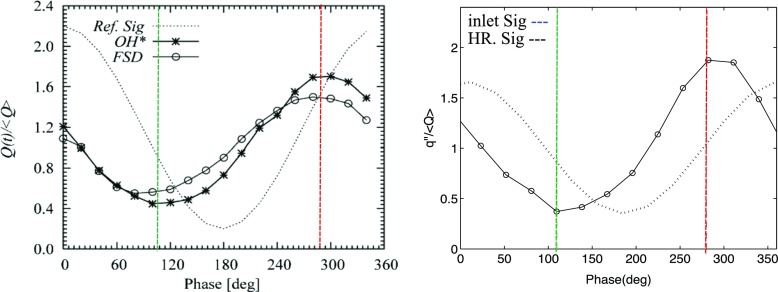
Integrated heat release at different phase angles for the experiment [10] (*left*) and LES (*right*)

Variations of the integrated heat release for inlet forcing amplitude *A* = 0.64 are presented in Fig. 14. The experimental data is shown in the left column and the LES results in the right column. For both plots, the inlet forcing (reference) signal is represented by dotted lines, and the integrated heat release signal is represented by solid lines with markers. It can be seen that the normalised amplitude and trend of the integrated heat release from LES match well with the experiment. In particular, the integrated heat release is found to attain the lowest value around 100–120° (dashed vertical green lines) for both experiment and LES, which corresponded to the condition where the flame surface area is at its lowest value due to the vortex rollup (Fig. 10, frame 2). The heat release subsequently increases and attains a peak value around 280–300° (dashed vertical red lines), where the flame lengthens and this corresponds to the highest flame surface area (Fig. 10, frame 5). This observation suggests that changes in the flame structure and dynamics at different phase angles have to be well predicted in LES to capture the FSD, and hence the global heat release response of the flame. It will be shown below that the ability to capture these localised changes is important to determine the saturation mechanism of the acoustically forced flame.

The dependence of the integrated heat release upon the inlet forcing amplitude at *f* = 160 Hz is represented in the form of a nonlinear FDF. The FDF is expressed as

14 $$ F(\omega,|u^{\prime}|)=\frac{Q^{\prime}/\overline{Q}}{u^{\prime}/\overline{U}}=G(\omega,|u^{\prime}|)e^{i \phi(\omega,|u^{,}|)} $$

where ${Q^{\prime }/\overline {Q}}$ is the normalised heat release rate fluctuation, ${u^{\prime }/\overline {U}}$ the normalised inlet velocity perturbation induced by the acoustic flow field. The FDF can also be expressed in the form represented by the gain *G*(*ω*,|*u*′|) and phase *ϕ*(*ω*, |*u*′|), which are functions of the frequency and amplitude. Such an expression for the FDF makes the assumption of weak non-linearity. This implies that the flame response to harmonic forcing is assumed to be primarily at the same frequency as the forcing signal, but with a gain and phase shift which depend upon the forcing amplitude as well as the frequency. The velocity at the combustor inlet contains a harmonic fluctuating component which takes the form shown in Eq. 12.

**Fig. 15:**
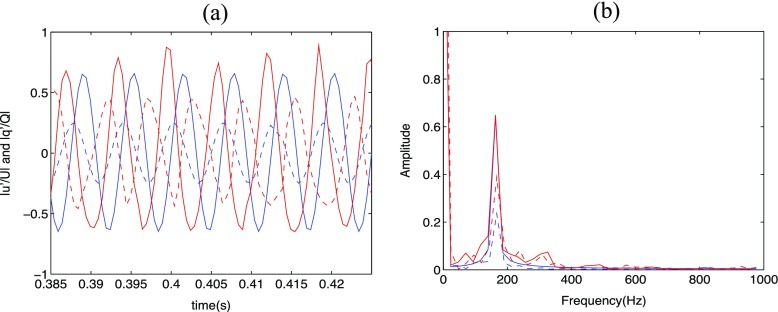
**a**: time series of the inlet velocity (*blue*) and integrated heat release (*red*) signal for A = 0.25 (*dashed lines*) and 0.64 (*solid lines*); **b** spectra of the corresponding signal

The forced flame produces a fluctuating heat release rate which is sampled during the simulations. The simulation results are based on at least 15 forcing cycles after the initial transients have disappeared. A Fast Fourier Transform (FFT) technique is used to transform the time series to the frequency domain. The FDF is extracted in Fourier space as the transfer function of the integrated heat release signal with respect to the inlet forcing velocity signal. The gain and phase of the FDF are obtained according to Eq. 14. Figure 15a shows the signals of the inlet velocity forcing (blue) and integrated heat release rate (red) for *A* = 0.25 and 0.64 from LES. It can be seen that the heat release rate is predominantly harmonic at the forcing frequency with some cycle-to-cycle variations. This observation is supported by the spectra of the signal in Fig. 15b where higher harmonics are not prominent, demonstrating that the nonlinear effects are indeed quite weak for the current configuration.

**Fig. 16:**
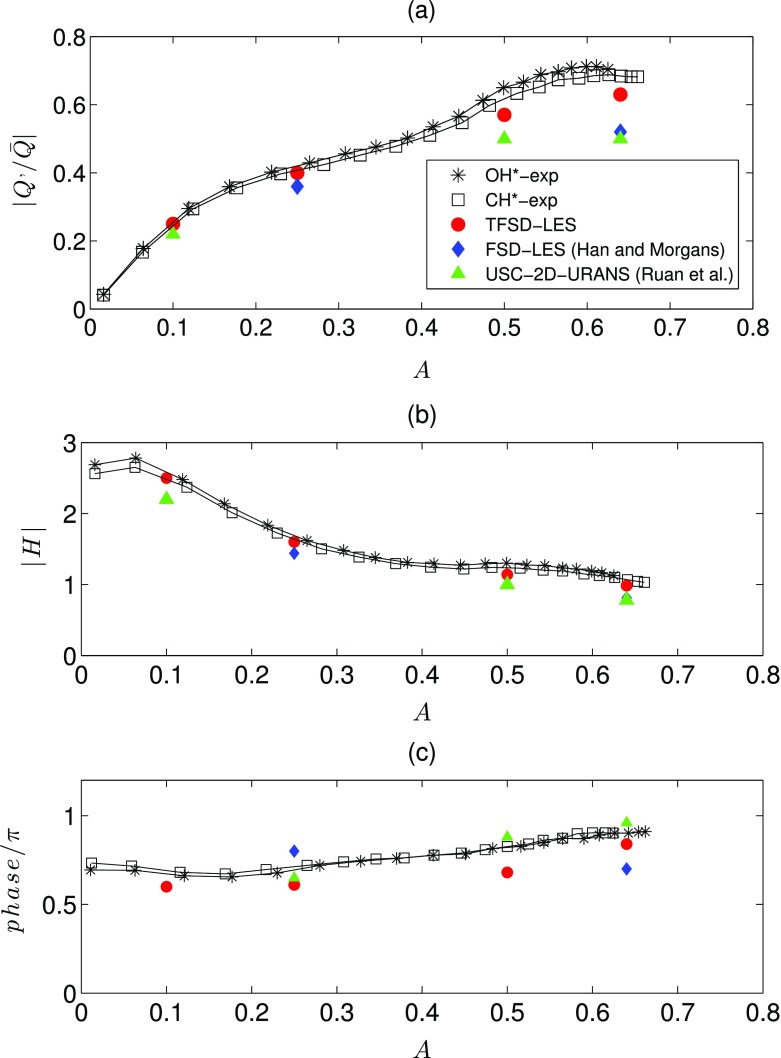
**a** Normalised integrated heat release and **b** gain of the FDF **c** phase of the FDF. Current LES (*red circle*); previous LES [23] (*blue diamonds*) and URANS [22] (*green triangle*)

The normalised amplitudes of the heat release rate fluctuation as a function of forcing amplitude, the FDF gain |*H*| and the FDF phase for *A* = 0.1, 0.25, 0.5 and 0.64 are shown in Fig. 16a, b and c respectively. Results computed from the current LES study (red circles), along with results than previous LES [23] (blue diamonds) and URANS [22] (green triangle) investigations are overlaid on the experimental curve from OH* and CH* chemiluminescence. Good agreement between the present LES predictions with experimental results is obtained for the FDF amplitude. The trend, including the linear region up to around *A* = 0.2, a transition region up to around *A* = 0.45 followed by a saturation region at *A* = 0.59, is evident. The time delay of the oscillation is also well captured, as evidenced by the phase of the FDF shown in Fig. 16c. At lower forcing amplitude *A* < 0.2 when the flame response is linear, both the current LES and previous numerical studies correctly reproduce the FDF gain from the experiment. Nevertheless, the current LES provides better predictions as compared to previous URANS [22] and LES results [23] especially at *A* = 0.64. This suggests that the ability of the TFSD approach to represent non-equilibrium effects on FSD transport is able to better capture the flame dynamics for saturation.

**Fig. 17:**
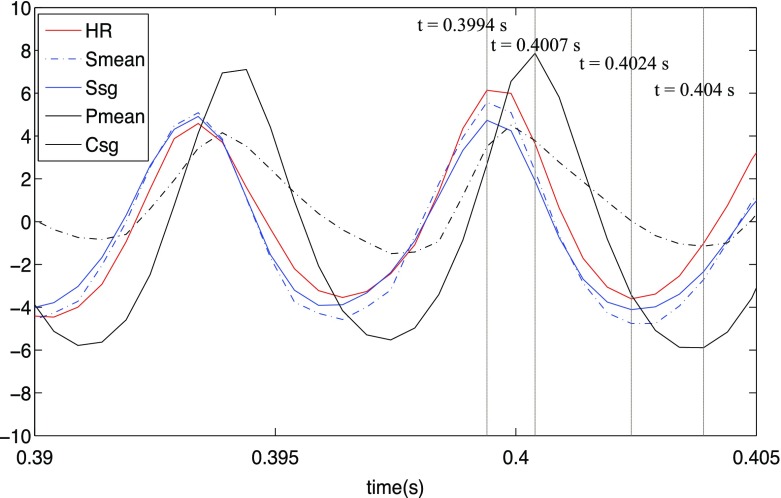
Spatially integrated heat release (*red*); resolved strain rate *S*_*mean*_ (*dotted blue line*); subgrid straining *S*_*sg*_ (*solid blue line*); resolved propagation and curvature *P*_*mean*_ (*dotted black line*) and subgrid curvature *C*_*sg*_ (*solid black line*)

The flame dynamics and underlying mechanisms leading to saturation are further investigated by analysing the results for *A* = 0.64. Figure 17 shows the normalised heat release and source terms in the TFSD equation which are integrated over the whole domain. The heat release is represented by the red lines. The terms responsible for FSD generation, including resolved strain rate *S*_*mean*_ and subgrid straining *S*_*sg*_ are represented by solid and dotted lines in blue, respectively. Conversely, the terms responsible for FSD destruction, including resolved propagation and curvature *P*_*mean*_ and subgrid curvature *C*_*sg*_ are denoted by solid and dotted lines in black, respectively. Here, we focus on four instances *t* = 0.3994, 0.4007, 0.4024 and 0404 s. The times 0.3994 s and 0.4024 s correspond to phase angles of maximum integrated heat release (phase angle = 300°) and minimum integrated heat release (phase angle = 120°). As the maximum value of *P*_*mean*_ and *C*_*sg*_ lag *S*_*mean*_, *S*_*sg*_ and the maximum heat release, the flame dynamics at 0.4007 s and 0.404 s are also investigated. Figure 17 shows that *S*_*mean*_ and *S*_*sg*_ have similar magnitude, but the contribution of *C*_*sg*_ exceeds *P*_*mean*_. It is also evident that the the integrated value of *C*_*sg*_ is lower (higher) than *S*_*sg*_ for the first (second) half of the oscillation cycle. This is primarily caused by small-scale annihilation of flame elements, reflected in the flame dynamics as shown below.

**Fig. 18:**
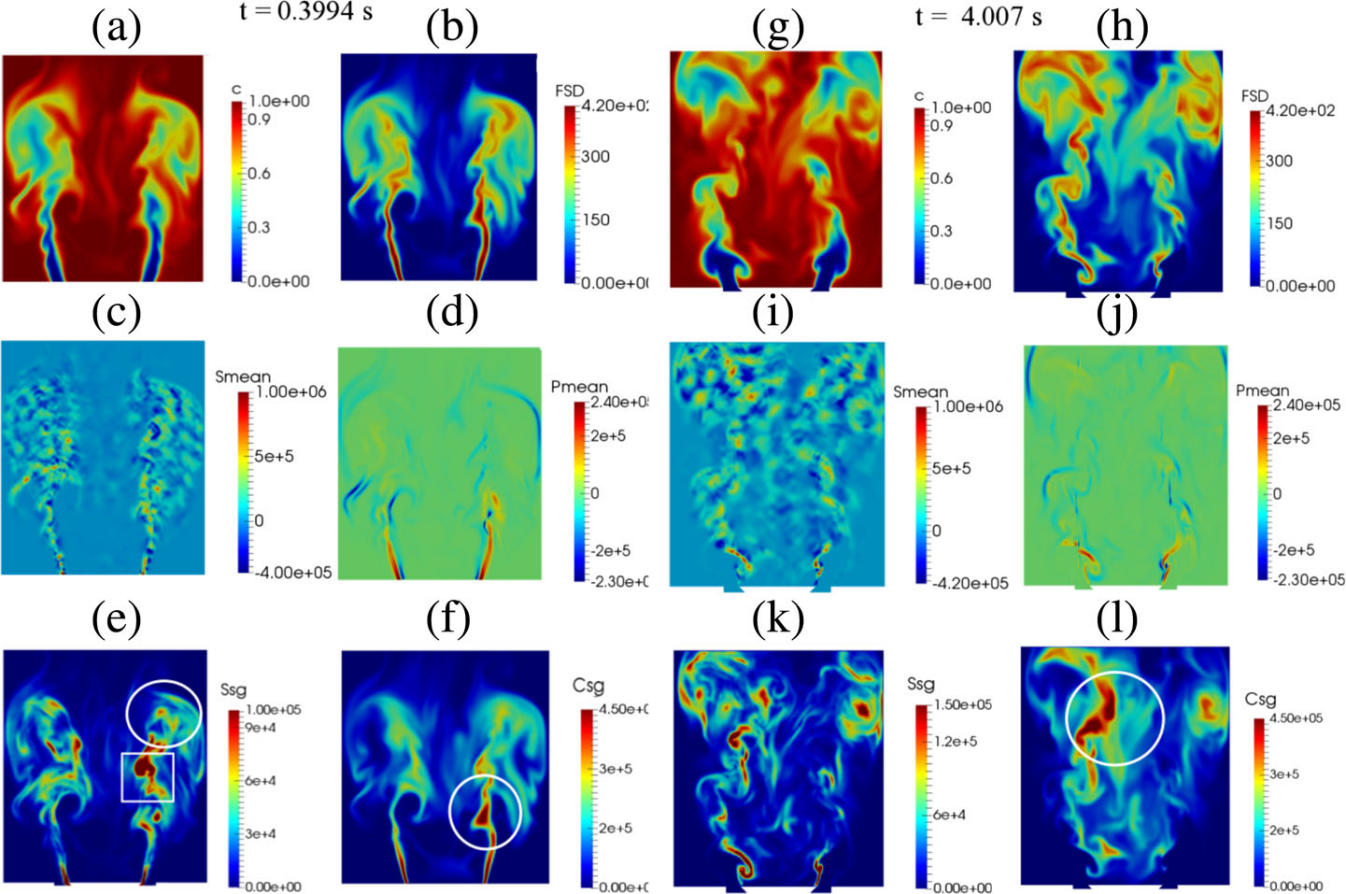
*Left two columns*: Progress variable (**a**), FSD (**b**), resolved straining (**c**), resolved propagation and curvature (**d**), subgrid straining (**e**) and subgrid curvature (**f**) at the phase angle of maximum integrated heat release rate at *t* = 0.3094 s. *Right two columns*: Progress variable (**g**), FSD (**h**), resolved straining (**i**), resolved propagation and curvature (**j**), subgrid straining (**k**) and subgrid curvature (**l**) at the phase angle of maximum integrated subgrid FSD at *t* = 0.4007 s

To describe the spatial distribution of each term in the FSD transport equation, snapshots of the instantaneous reaction progress variable, FSD, resolved straining, resolved propagation and curvature, subgrid straining and subgrid curvature at different instances are presented. Figure 18 shows these quantities at *t* = 0.3994 s (left two columns, Fig. 18a–f) and at *t* = 0.4007 s (right two columns, Fig. 18g–l). For *t* = 0.3994 s, the globally integrated heat release is at its maximum. The progress variable (Fig. 18a) and FSD (Fig. 18b) contours indicate lengthening of the flame and formation of vortices close the side walls of the combustion chamber. Pockets of reacting gas begin to pinch-off from the flame sheet. Large variations in resolved strain rate (Fig. 18c) and resolved propagation and curvature (Fig. 18d) can be seen throughout the burner, particularly in the shear layer and downstream vortices. As shown in Fig. 18e, localised regions with high subgrid strain can be seen close to the inner shear layer consistent with where pinch-off occurs (indicated by the white square) and in the downstream region of the flame when subjected to intense rollup (indicated by the white circle). Figure 18f shows that subgrid curvature is prominent where interaction and collapse of flame elements take place (indicated by the white circle).

At *t* = 0.4007 s (Fig. 18g–l), the progress variable (Fig. 18g) and FSD (Fig. 18h) contours depict a significant shortening of the flame as a new vortex forms, and the downstream flame sheet has now completely detached from the upstream branch and convects downstream. Fragmented flame elements can be seen downstream where the FSD is high. Variations in resolved strain rate (Fig. 18i) and subgrid straining (Fig. 18k) are observed close to the bluff body lip, but become prominent towards the wake where vortices are present. At the downstream region, high magnitude of localised subgrid curvature can be seen due to small-scale destruction of flame elements (indicated by the white circle). This behaviour is consistent with the earlier observation where the globally integrated FSD destruction term *C*_*sg*_ is at its maximum (Fig. 17).

**Fig. 19:**
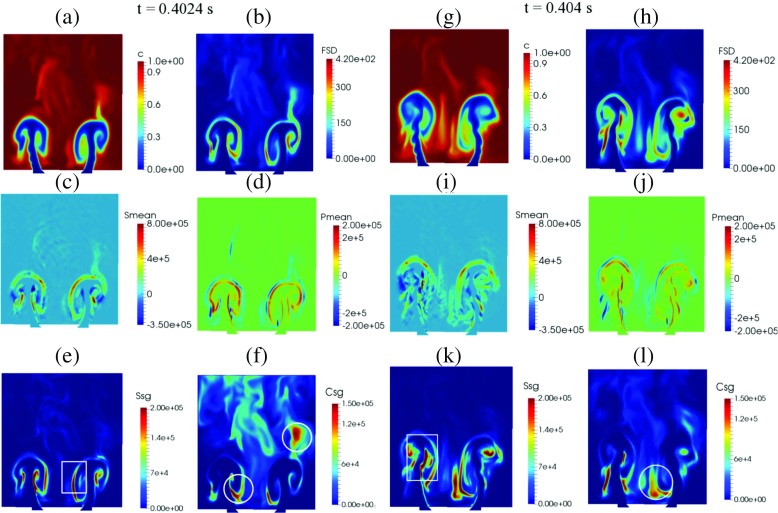
*Left two columns*: Progress variable (**a**), FSD (**b**), resolved straining (**c**), resolved propagation and curvature (**d**), subgrid straining (**e**) and subgrid curvature (**f**) at the phase angle of minimum integrated heat release rate at *t* = 0.4024 s. *Right two columns*: Progress variable (**g**), FSD (**h**), resolved straining (**i**), resolved propagation and curvature (**j**), subgrid straining (**k**) and subgrid curvature (**l**) at the phase angle of minimum integrated subgrid FSD at *t* = 0.404 s

Figure 19 shows the same quantities at *t* = 0.4024 s (left two columns, Fig. 19a–f) and at *t* = 0.404 s (right two columns, Fig. 19g–l ). For *t* = 0.4024 s, the integrated heat release is lowest. Strong rollup of the flame front and formation of cusps in the inner shear layer can be observed (Fig. 19a). High FSD (Fig. 19b) is present along the regions of the flame wrapped by counter-rotating vortices. The resolved strain rate (Fig. 19c) and resolved propagation and curvature (Fig. 19d) terms become more localised along the shear layer of the distorted flame sheet as it is entrained by the vortex. As shown in Fig. 19e, the flame experiences high strain rate at the edges of the vortex when it is aligned with the flow direction (indicated by the white square). Values of the subgrid curvature term (Fig. 19f) are much higher compared to the subgrid straining term of the flame where rollup occurs and cusps are present (indicated by the white circle), suggesting that flame annihilation is dominant locally due to interaction and collapse of flame elements that are entrained by the vortex. Subgrid curvature remains high in the wake region downstream.

At *t* = 0.404 s, the integrated subgrid FSD destruction term is at its minimum. The flame starts to lengthen (Fig. 19g) as the vortex convects downstream. Also noticeable is that the flame is slightly lifted in the outer shear layer. High FSD (Fig. 19h) is observed at the edges of the shear layers where the outer and inner flame branches collapse onto a single flame sheet. This is reflected by the high resolved strain rate (Fig. 19i) and resolved propagation and curvature (Fig. 19h) terms in these regions. The subgrid strain rate (Fig. 19k) increases as the extent of rollup increases as the flame is entrained by the convected vortex formed at the flameholder lip (indicated by the white square). High subgrid curvature (Fig. 19l) is present particularly in the regions where the flame collapses in the inner shear layer (indicated by the white circle).

As seen previously, the magnitude of global heat release depends on the bulk location where the FSD is generated or destroyed. When the integrated heat release is highest, the generation or destruction of flame surface area occurs throughout the combustor (Fig. 18). Conversely, when the integrated heat release is close to its lowest value, these activities tend to concentrate in regions closer to the bluff body (Fig. 19). Also, the flame destruction is dominant in the wake. This is confirmed by the high localised *C*_*sg*_ in the downstream regions seen in Figs. 18l and 19f, which corroborates the higher value of integrated *C*_*sg*_ than *S*_*sg*_ between *t* = 0.4007 to 0.4024 s in Fig. 17. Lastly, the fact that the outer shear layer flame is lifted suggests that the saturation flame dynamics may be affected by it, as observed in previous experimental work [14]. This behaviour deserves further study.

**Fig. 20:**
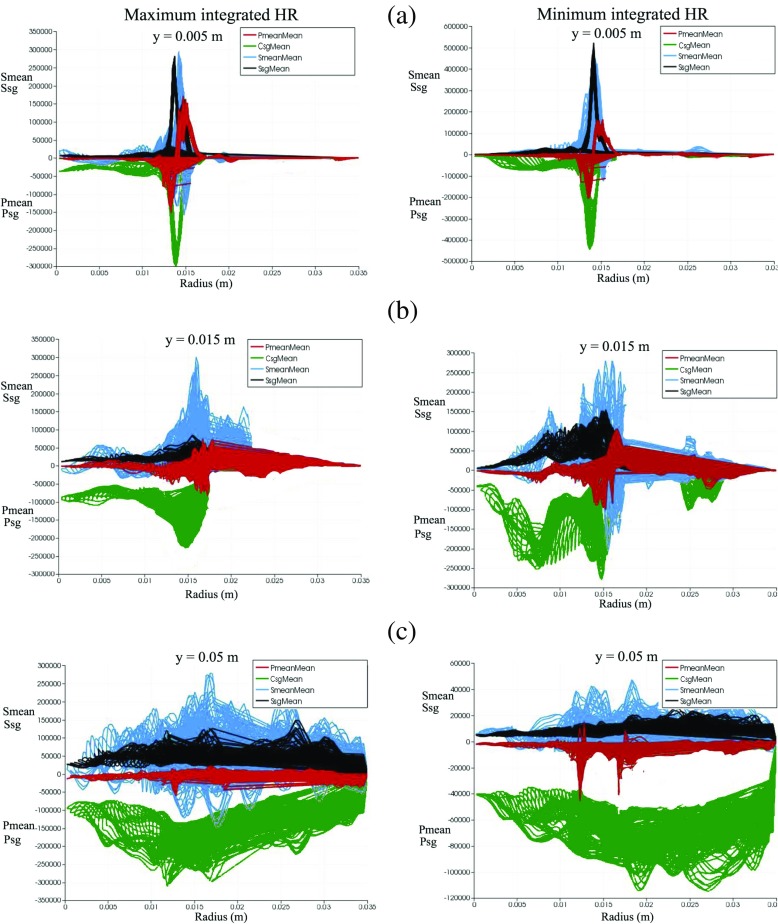
Phase-averaged values of resolved straining (*blue*) resolved propagation and curvature (*red*) subgrid straining (*black*) and subgrid curvature (*green*) terms from the TFSD model plotted in **a** plane 1, **b** plane 2 and **c** plane 3 for phase angle of maximum (*left column*) and minimum (*right column*) integrated heat release. Plane 1 corresponds to y = 0.005 m (indicated by dotted white line in Fig. 5a), plane 2 corresponds to y = 0.015 m (indicated by solid white line in Fig. 5a) and plane 3 corresponds to y = 0.05 m (indicated by dashed white line in Fig. 5a)

The plots of each phase-averaged terms in the FSD transport equation along the radial distance from the centreline at plane 1 (shear layer y = 0.005 m), plane 2 (rollup/stretched region y = 0.015 m) and plane 3 (downstream region y = 0.05 m) are presented in Fig. 20. Results corresponding to phase angles of maximum and minimum integrated heat release are presented in the left and right columns, respectively. At plane 1, similar trends can be seen for both cases. The subgrid strain rate (black) and subgrid curvature (green) terms are found to be in equilibrium. It can also be observed that the resolved tangential strain rate (blue) and resolved propagation and curvature (red) terms attain both positive and negative values, suggesting that each term can either generate or destroy FSD in the shear layer close to flameholder lip.

At plane 2, the magnitude of the strain rate term becomes higher in the resolved-scale (blue) than in the subgrid-scale (black) for both cases. This demonstrates that the resolved FSD generation tends to become dominant towards the rollup region due to the formation of large-scale vortices and their interactions with the flame. The subgrid curvature (green) term is also much larger compared to other terms. More importantly, there is a distinct difference for the subgrid curvature (green) term between the two cases. The value of subgrid curvature term at the phase angle of minimum integrated heat release (Fig. 20, right column) is much higher especially at the inner shear layer close to the centreline indicated by radius *r* = 0.005–0.015 m. In these regions, cusps are formed due to rollup of the flame into the central recirculation zone and collapse of flame elements (refer to Fig. 19f). This demonstrates that contrary to expectations that flame area might be increased by the flame elements wrapping around the vortex, the FSD has decreased due to flame surface area destruction at small scales and the formation of cusps in the tip of the rollup. This causes a very rapid reduction in the flame area, and therefore in the heat release rate. As a result, the flame heat release response does not increase proportionately with the perturbation, and saturation occurs. Such a behaviour was not observed however for the phase angle with maximum integrated heat release (Fig. 20, left column). Instead, the resolved tangential strain rate (blue) balances the subgrid curvature (green) term mainly in the shear layer. This suggests that for this phase angle, the contribution to FSD generation at the resolved scales is higher at this phase angle due to unsteady vortices that are generated in the two adjacent shear layers and then roll up into larger structures in the region between the inner and outer recirculation zones.

At plane 3, destruction of flame surface area at the subgrid scales is found to be dominant for both cases, as indicated by the values of subgrid curvature (green) that are much higher compared to subgrid straining (black). This behaviour is consistent with the mechanisms observed in Figs. 18f and 19f, respectively. The extent of resolved straining (blue) is again higher for the phase angle with the maximum integrated heat release rate. Lastly, it should be noted that a general trend is observed for both forced and unforced cases, where the local balance between subgrid FSD generation and destruction is evident in the shear layer (y = 0.005 m) but subgrid FSD destruction becomes increasingly dominant towards the downstream region of the flame (y = 0.05 m).

## Conclusions

LES of a turbulent bluff-body stabilised flame was performed under unforced and forced conditions. Validation of both the unforced cold flow and reacting flow was conducted. The mean and rms axial velocity profiles were compared for the cold flow, whereas the instantaneous OH-PLIF and FSD measurements were used to validate the reacting flow. The predicted flame shape and position are in good agreement with the experiment. The TFSD model was shown to be able to describe non-equilibrium effects of flame surface evolution. In particular, the subgrid strain and subgrid curvature were found to be in equilibrium in the shear layer, but the magnitude of the subgrid curvature was much higher in the downstream region.

Simulations of forced reacting flows were then conducted at four conditions corresponding to forcing amplitude *A* = 0.1, 0.25, 0.5 and 0.64 at forcing frequency 160 Hz to describe the saturation behaviour observed in the experiments. The phase-averaged FSD results were compared against the experiment, showing that changes in the flame dynamics throughout the forcing cycle were well described. Notably, the rollup of the flame front due to the growth and convection of the vortices, as well as the elongation of the flame and interaction with the wall were clearly observed. The agreement of the gain and phase of the computed FDF with the experiment was good, and the improved prediction obtained in the current work compared to previous URANS and LES results especially at high forcing amplitudes demonstrated that accurate representation of the local variations of the flame structure is necessary to determine the global heat release response. The mechanisms leading to the saturation of the flame response at *A* = 0.64 were also studied, and it was found that localised regions of flame area generation and destruction vary between phases of the forced oscillation. In particular, at the phase angle where the integrated heat release was at its maximum, pinch off of the flame, rollup of the flame front and flame annihilation due to flame elements interaction were all present. These types of behaviour were highlighted by localised regions with high subgrid straining and subgrid curvature, suggesting that the balance between flame area production and destruction governed the global heat release modulation. The flame was also found to be subjected to high resolved tangential strain rate in the downstream region due to large-scale vortices. In contrast, a clear shortening of the flame and intense rollup of the flame front were observed at the phase angle with lowest integrated heat release. It was found that the subgrid curvature was dominant in regions where cusps were formed and in the far-wake regions where flame annihilation was present. The current LES study supports the experimental observation that flame surface area modulation plays a major role in determining the heat release magnitude. The transition to a nonlinear behaviour coincides with the shedding of vortices from the lip of the bluff body and the resultant distortion of the flame sheet as the inner and outer flames collapse, leading to the destruction of flame area.
